# Plant Diversity Changes during the Postglacial in East Asia: Insights from Forest Refugia on Halla Volcano, Jeju Island

**DOI:** 10.1371/journal.pone.0033065

**Published:** 2012-03-16

**Authors:** Jiri Dolezal, Jan Altman, Martin Kopecky, Tomas Cerny, Stepan Janecek, Michael Bartos, Petr Petrik, Miroslav Srutek, Jan Leps, Jong-Suk Song

**Affiliations:** 1 Institute of Botany, Academy of Sciences of the Czech Republic, Pruhonice, Czech Republic; 2 Department of Botany, Faculty of Science, University of South Bohemia, Ceske Budejovice, Czech Republic; 3 Department of Biological Science, College of Natural Sciences, Andong National University, Andong, Republic of Korea; Lakehead University, Canada

## Abstract

Understanding how past climate changes affected biodiversity is a key issue in contemporary ecology and conservation biology. These diversity changes are, however, difficult to reconstruct from paleoecological sources alone, because macrofossil and pollen records do not provide complete information about species assemblages. Ecologists therefore use information from modern analogues of past communities in order to get a better understanding of past diversity changes. Here we compare plant diversity, species traits and environment between late-glacial *Abies*, early-Holocene *Quercus*, and mid-Holocene warm-temperate *Carpinus* forest refugia on Jeju Island, Korea in order to provide insights into postglacial changes associated with their replacement. Based on detailed study of relict communities, we propose that the late-glacial open-canopy conifer forests in southern part of Korean Peninsula were rich in vascular plants, in particular of heliophilous herbs, whose dramatic decline was caused by the early Holocene invasion of dwarf bamboo into the understory of *Quercus* forests, followed by mid-Holocene expansion of strongly shading trees such as maple and hornbeam. This diversity loss was partly compensated in the *Carpinus* forests by an increase in shade-tolerant evergreen trees, shrubs and lianas. However, the pool of these species is much smaller than that of light-demanding herbs, and hence the total species richness is lower, both locally and in the whole area of the *Carpinus* and *Quercus* forests. The strongly shading tree species dominating in the hornbeam forests have higher leaf tissue N and P concentrations and smaller leaf dry matter content, which enhances litter decomposition and nutrient cycling and in turn favored the selection of highly competitive species in the shrub layer. This further reduced available light and caused almost complete disappearance of understory herbs, including dwarf bamboo.

## Introduction

Understanding how past climate changes affected biodiversity is a key issue in contemporary ecology and conservation biology. These diversity changes are, however, difficult to reconstruct from paleoecological sources alone, because macrofossil and pollen records do not provide complete information about species assemblages. There are relatively precise fossil data on the diversity of trees, which is linked to the ability of long-distance pollen dispersal in many tree genera [1], a high level of deposition and good preservation of woody macro-remains [2]. Much less information is available for herbaceous species because their pollen identification to species level is usually impossible [3], pollens of insect-pollinated herbs are locally dispersed and hence underestimated in pollen records, and the limited amount of well-preserved herb macrofossil deposits. Unlike tropical forests, where woody species constitute the major component of plant diversity [4], herbaceous plants predominate in temperate and boreal forests in terms of species richness [5]. Hence, excluding herbaceous species from paleoenvironmental assessments could lead to misleading conclusions about the vascular plant diversity changes.

Ecologists therefore use information from modern analogues of past communities in order to get a better understanding of past diversity changes [6], [7]. These analogues are especially useful when they represent direct relicts from past communities surviving *in situ* – e.g. because of high environmental heterogeneity. While modern analogues of glacial and postglacial forests have been studied in Europe, North America and North Asia [6], [7], [8], [9], [10], East Asia remains largely unexplored.

Modern analogues of the late glacial and postglacial forests can be found on the East Asian oceanic islands, previously connected with the mainland during the Last Glacial Maximum (LGM, 21–18 ka BP), and then separated following postglacial sea transgression during the Late Glacial – Holocene transition (14–11 ka BP) [11]. Jeju Island is one of them, harboring forest refugia that correspond to the temporal sequence in which different forest communities replaced each other during the postglacial [12]. These forests nowadays encompass separate altitudinal belts along the slopes of Halla Volcano, which reach an altitude of almost 2000 m a.s.l. and represent high habitat heterogeneity in a relatively small area [13]. During the LGM, shallow epicontinental seas between China, Korea and Japan dropped by about 130 m [14], exposing extensive continental shelves and connecting Jeju Island with the mainland [15]. This enabled floristic exchange between Jeju Island, mainland Korea and China via land-bridges [16], along with a migration of plant species of subtropical origin from southern Japan (Kyushu Island) and Taiwan [17].

Jeju Island was virtually on a crossroads of several migration routes, bringing together different floristic elements including arctic-alpine, temperate forest, steppe as well as subtropical ones [12]. Macrofossil and palynological records suggest that under the cold and dry climate of the full- and late-glacial, lowland areas of Jeju Island were covered by forest-steppes with woodland refugia of deciduous broad-leaved trees, while upland areas were covered by tundra with patches of conifer trees such as *Abies, Picea, Pinus* and *Taxus* [18], [19]. *Artemisia*-dominated cold steppe vegetation was widespread in more continental areas towards mainland Korea and China [20]. In the oceanic southern parts glacial refugia of temperate broad-leaved forests were preserved [18]. The oceanic climate on Jeju Island [11], with a relatively small annual temperature amplitude and abundant moisture, ensured continuous presence of deciduous broad-leaved trees (e.g. *Quercus* spp.) during the LGM (ca. 21.8 to 14.4 ka BP) [18]. Following the climatic amelioration of the early Holocene, these woodlands expanded at the expense of open steppe, and were gradually invaded by warm-temperate broad-leaved deciduous hornbeam (*Carpinus* spp.) and evergreen subtropical trees (e.g. *Camellia* spp., *Daphniphyllum* spp.) after ca. 12 ka BP [21].

Increasing temperature and humidity during the early Holocene induced upward migration of species from hornbeam and oak woodlands [22], resulting in reduction or total disappearance of coniferous forests and arctic-alpine tundra vegetation from most Korean mountains as they do not reach sufficiently high elevations. This transition from late-glacial open conifer woodlands to mid-Holocene closed-canopy oak-hornbeam forests was a major environmental change, which probably had a significant effect on biodiversity.

In this paper we aim to provide new insights into these changes through a comparative study of different forest types occurring on Halla Volcano of Jeju Island. We try to elucidate how vegetation, species diversity and environmental variables have differed between these forest types using multidimensional fuzzy set ordination and species rarefaction curves. We focused on the diverse range of vascular plants present (trees, shrubs, herbs, lianas, ferns etc). All of these are important for proper forest ecosystem function and yet they occupy their own niche [7]. We attempt to explain the postglacial species replacement by studying life-history strategies of dominant woody species, including their mode of regeneration, growth and mortality rate, and functional traits related to resource acquisition.

## Materials and Methods

### Study area

**Figure 1:**
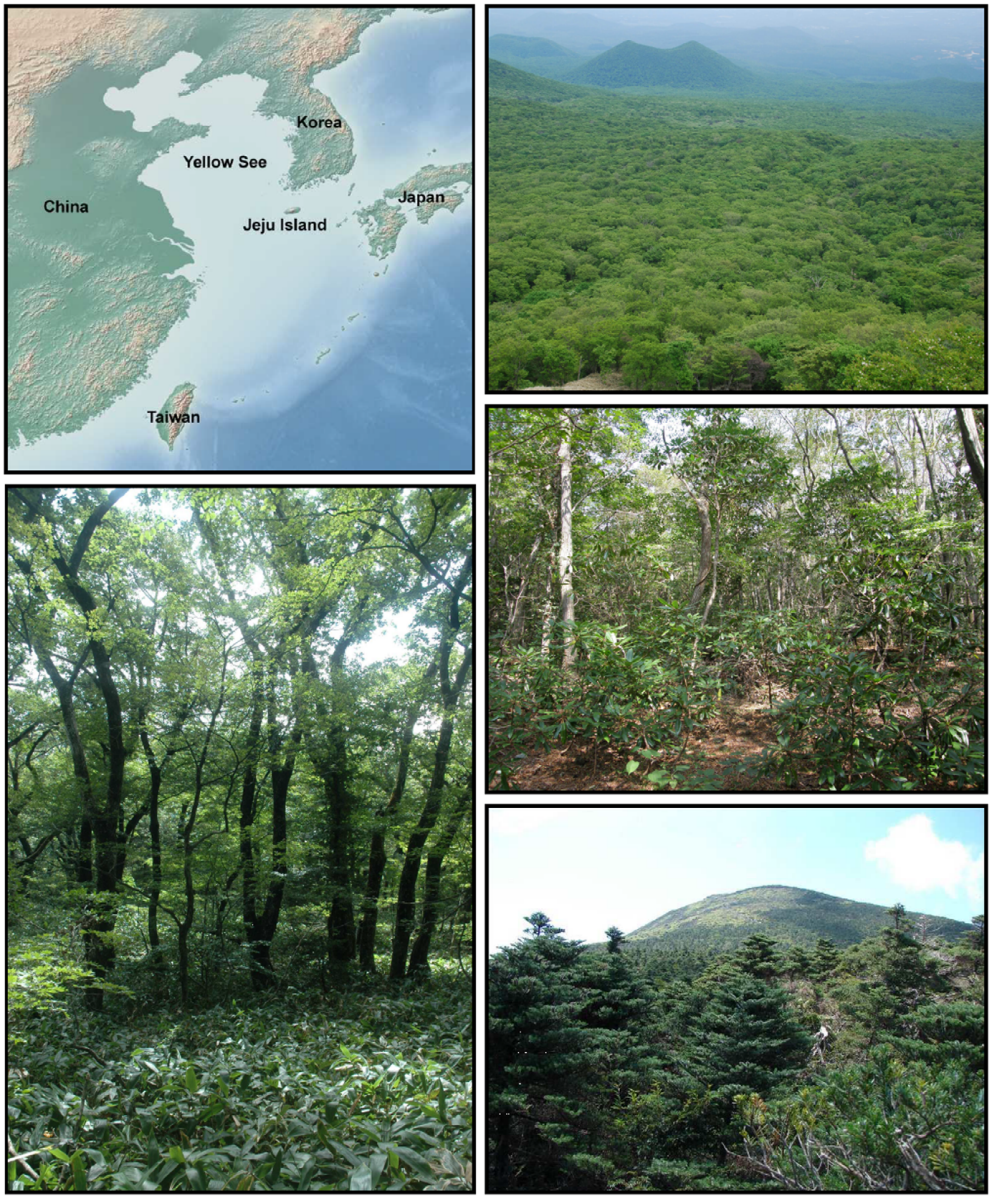
Location of Jeju Island in NE Asia (top left picture). Studied oak-hornbeam forests on the south-east slope of Halla Volcano between 500 and 1200 m a.s.l. (top right). Closeup view of the *Carpinus* forests with sub-canopy evergreen trees (*Daphniphyllum macropodum*) at 750 m (middle right). *Quercus* forest with dwarf-bamboo (*Sasa quelpaertensis*) understory at 1200 m (bottom left). Westward view of Halla Volcano with subalpine *Abies koreana* forests and grassland around the top (bottom right).

The research was conducted in Hallasan National Park on Jeju Island, the largest Korean island (33°10′ ≈ 33°34′ N, 126°10′ ≈ 127° E), 90 km south of the Korean Peninsula (Figure 1). Hallasan National Park (153 km^2^) is situated in the centre of the island around an extinct Halla Volcano, the highest peak in South Korea (1,950 m a.s.l.), constructed on the continental shelf of the Yellow Sea. The mountain was formed in the Middle Pleistocene, about 780 ka ago, and was an active volcano until about 25 ka BP. It is mostly composed of basalts covered by andisols. The climate on the island is strongly affected by winter cold air masses from Siberia, and summer monsoons and tropical storms (typhoons) from the Pacific Ocean. The northeastern coast of the island (Jeju City, data from 1978–2007) has a mean annual temperature of 15.7°C, mean January temperature 5.8°C, mean July temperature 26.7°C, and only 17 days per year with temperatures below 0°C. Mean annual temperature at Orimok weather station, at 970 m a.s.l., is 9.7°C, and that of the subalpine zone is estimated to be 4.5 to 7.3°C, based on Mt Halla’s temperature lapse rate of 0.58°C/100 m [23]. The summer monsoon brings abundant moisture from the ocean and produces heavy rainfall. Precipitation rises from about 1500 mm in coastal areas to over 4000 mm in upland areas [24].

As a consequence of the extensive areas (153 km^2^) of natural vegetation, several schemes have identified Halla Volcano and its forests as a global priority region for conservation. Recently, Hallasan National Park has been designated an UNESCO Biosphere Reserve (2002) and a World Heritage Site (2007) for its pristine environments, unique altitudinal zonation of vegetation and high level of endemism [12].

### Data collection

The field data were collected yearly between 2006 and 2011. The time of sampling corresponded with the peak of the vegetation season, which lasts from June to mid-September in the area. All necessary permits were obtained for the field studies described below from the authorities of Hallasan National Park.

The composition of three forest types, occupying an area of ∼ 90 km^2^ within Hallasan Natural Reserve, was studied (Figure 1). These distinguished forest types corresponded to the temporal sequence in which dominant tree species replaced each other in the late-glacial and mid-Holocene and today forming three altitudinal vegetation zones [13], as follows: (1) *Carpinus* (47 plots studied): warm-temperate forests at lower elevations (500–1,000 m) dominated by *Carpinus laxiflora, C. tschonoskii,* and sub-canopy evergreen trees (*Daphniphyllum macropodum*), (2) *Quercus* (43 plots studied): cool-temperate deciduous broad-leaved forests at middle elevations (1000–1400 m), dominated by oak (*Q. mongolica*) and maple (*Acer pseudosieboldianum*), (3) *Abies* (56 plots studied): mountain-temperate conifer forests at higher elevations (1400–1900 m), dominated by fir (*A. koreana*) and, less frequently, by birch (*Betula ermanii*) (Table 1).

**Table 1:** Comparison of abiotic (altitude, topographical indices and soil physico-chemical parameters) and biotic variables (species richness of different life-forms) between three forest types of Halla Volcano, South Korea.

	Forest type		
	Carpinus	Quercus	Abies
Number of plots 100 m^2^	47	43	56
Altitude (m a.s.l.): mean	846	1223	1638
Radiation (topographical index)	0.95^a^	0.93^a^	0.96^a^
Heat (topographical index)	0.96^a^	0.92^a^	0.95^a^
Slope (°)	9.6^a^	11.4^ab^	12.6^b^
Cover of exposed rock (%)	23^a^	8^b^	26^ a^
Depth of the Ah horizon (cm)	17^a^	35^ab^	39^b^
Gravel in Ah horizon (%)	0.05^a^	0.32^a^	0.77^b^
Soil pH (actual)	4.3^a^	4.3^a^	4.5^a^
C organic (g kg^–1^)	166^a^	215^a^	238^b^
N total (g kg^–1^)	16.4^a^	17.1^a^	12.8^a^
NH_4_^+^-N (mg kg^–1^)	135^ab^	220^a^	94^b^
NO_3_^–^-N (mg kg^–1^)	24^a^	25^a^	7^b^
PO_4_^–3^-P (mg kg^–1^)	18.3^a^	33.3^b^	24.7^ab^
Mg^2+^ (mg kg^–1^)	215^ab^	273^a^	185^b^
Ca^2+^ (mg kg^–1^)	410^b^	958^a^	499^b^
Total richness of vascular plants	189	149	202
No. of all plant species per 100 m^2^	27.8^a^	19.1^b^	34.2^a^
No. of woody species	14.9^a^	10.3^b^	11.1^a^
No. of herb species	12.8^a^	8.8^b^	23.1^c^
No. of deciduous broadleaved trees	8.4^a^	6.2^b^	3.7^c^
No. of evergreen broadleaved trees	0.9^a^	0.4^b^	0.2^c^
No. of conifer trees	0.7^a^	0.9^b^	2.1^c^
No. of deciduous shrubs	2.8^a^	1.6^b^	5.1^c^
No. of evergreen shrubs	0.9^a^	0.5^b^	0.2^c^
No. of lianas	4.1^a^	2.1^b^	1.8^b^
No. of ferns	1.6^a^	1.4^a^	5.2^b^
No. of forbs	6.8^a^	4.9^a^	12.6^b^
No. of sedges	0.5^a^	0.3^a^	2.1^c^
No. of grasses	1.2^a^	0.9^b^	1.3^a^
Cover of tree layer (%)	87^a^	86^a^	67^b^
Cover of shrub layer (%)	35^a^	26^b^	36^ab^
Cover of herb layer (%)	81^a^	87^a^	67^b^
Cover of dwarf bamboo (%)	58^a^	82^b^	34^c^

Means sharing the same lowercase superscript letter are not significantly different at *P* < 0.05 (ANOVA followed by Tukey post-hoc tests). Variables without superscript letters were not tested.

For each forest type, the plant composition was studied in 100 m^2^ square plots of homogeneous mature vegetation (with trees older than 150 years and no sign of logging). Strongly disturbed forests and low altitude pine plantations were avoided. In each plot we recorded all species of vascular plants (taxonomy and nomenclature according to [25]), and estimated their individual covers and the total percentage covers of the herb (< 1 m high), shrub (1–5 m) and tree (> 5 m) layers. Since we were not able to visit all areas (botanical survey in the park became highly restricted after the UNESCO designation), we supplemented our records with data collected by Song & Nakanishi [26] and Yim et al. [13]. In total, we gathered vegetation data for 146 sites, including 313 taxa of vascular plants, belonging to 190 genera and 92 families. Altitude was measured with a Garmin 60CSx GPS receiver (Garmin International Inc., Olathe, Kansas, USA). Slope inclination was measured with a clinometer (Haglöf Hypsometer, Sweden). Topographical indices of radiation and heat were calculated from latitude, slope inclination and aspect, according to equation 3 in McCune & Keon [27].

In order to explore possible changes in soils during the postglacial forest development, we collected soil samples for chemical analyses in subsample of 44 vegetation plots. These were selected to represent a habitat range similar to that of the full sample set of plots. Within each plot, a mixed sample consisting of eight systematically distributed subsamples of soil was collected from the *Ah* horizon. The collected soil was air-dried, and sieved through a 2 mm mesh. The remaining soil gravel was weighed and removed. The following standardized analyses were performed: soil reaction (active in water solution and exchangeable in KCl solution), total carbon and nitrogen content, NH_4_^+^-N, NO_3_^–^-N, PO_4_^–3^-P (Mehlich-3) content, exchangeable calcium and magnesium content in ammonium acetate solution. Additionally, the soil profile in a pit dug next to the studied plot was described and the thickness of soil horizons measured.

In the search for mechanisms responsible for the postglacial species replacement, we explored differences in stand structure and life-history strategies of dominant woody plants between forest types. We collected information on specific leaf area (SLA), leaf dry matter content (LDMC), and leaf tissue nitrogen and phosphorus (LNC, LPC) concentrations for 44 dominant woody species. These traits have been proved to be important indicators of plant strategies reflecting a fundamental trade-off in plant functioning between a rapid production of biomass (high SLA, high foliar N, low LDMC) and efficient conservation of nutrients (low SLA, low foliar N, high LDMC). We used a single rope technique and 3-m long pruning shears to collect 15–20 leaves from the upper, well-lit crown regions (3 to 5 individuals per species, in total 3475 leaves from 191 trees randomly selected at different elevations). Fresh leaf blades were weighed and photographed in the field to assess LDMC on fresh-weight basis and SLA (leaf area per unit dry weight), respectively. Dry leaves were weighed individually, combined by tree individuals, ground into powder, and analyzed colorimetrically for N and P content using an FIA star 5010 Analyzer (Foss Tecator AB, Höganäs, Sweden).

Due to field-time restrictions (overnight stay in the park is forbidden), stand structural data were collected only on the eastern slopes of Halla Volcano using a system of 16 permanent plots. We established five plots in *Carpinus* forests (total area of 1.62 ha), five plots in *Quercus* forests (total area of 1.25 ha), and seven plots in *Abies* forests (total area of 0.28 ha). Each live and dead tree within the plot was identified, marked and its position (x,y coordinates), regeneration mode (multi-stemmed polycormons vs. single stems), diameter at breast height (DBH, at 1.3 m), and height were recorded. In total, we measured 10755 trees >1.3 m tall. Furthermore, we extracted wood cores from 1650 randomly selected trees at the 0.8 m above ground with a steel borer (Mora, Sweden) to age the trees and to reconstruct their growth histories. Tree-rings were counted from pith to bark and their widths measured to the nearest 0.01 mm using the TimeTable measuring device and PAST32 software (http://www.sciem.com).

To assess shading potential of tree species, we quantified light transmitted through the canopy from hemispherical photographs taken at 1.8 m above ground at randomly selected points within each plot (10–20 points depending on the plot size; in total 200 photographs). A Nikon F9 digital camera with Nikkor fisheye lens was used. The obtained photographs were converted to black–and–white bitmaps using automatic thresholding implemented in SideLook 1.1 [28]. Transmitted direct, diffuse, total solar radiation, and canopy openness were calculated in the Gap Light Analyzer software [29].

### Data analysis

To explore patterns in species composition, we performed non-metric multidimensional scaling (NMDS) on a Bray-Curtis dissimilarity matrix calculated from square root transformed percentage cover data standardized by sample totals. We ran NMDS in two dimensions and used several random starts in order to achieve the optimal configuration. The inspection of NMDS results and our field knowledge led us to the hypothesis that elevation and cover of dwarf bamboo (*Sasa quelpaertensis*) are the primary drivers of vegetation patterns on Halla Volcano, resulting in formation of three basic forest types as described above. Therefore, we tested effects of elevation and bamboo cover on vegetation composition directly through multidimensional fuzzy set ordination (MFSO) [30]. MFSO is a robust and highly effective ordination method, which incorporates environmental variables directly into calculation of ordination axes and tests their relationship with vegetation dissimilarity through permutation [30]. A goodness of fit criterion in MFSO is the correlation between Euclidean distances of all samples in the ordination space and their original dissimilarities. High correlation means effective ordination in which environmental variables have a large effect on species composition [30].

MFSO is quite a new constrained ordination method, which substantially differs from other widely used methods (e.g. CCA, db-RDA) [31]. Therefore, we performed additional tests and compared configuration of samples in MFSO with their configuration in NMDS. For comparison, we used the symmetric Procrustes analysis and tested its significance by permutation [32]. The result of this analysis shows the efficiency of MFSO constrained by environmental variables in comparison to the best possible configuration of plots in the same number of dimensions achieved by NMDS. All multivariate analyses were performed within R 2.12 [33] with the fso [34] and vegan [35] packages.

In order to test whether the species composition differs between the three forest types, we used Multi Response Permutation Procedure (MRPP) [36], calculated on the same dissimilarity matrix as was used in NMDS. Plant species composition of the three forest types was summarized in a synoptic table, in which positive diagnostic (indicator) species were determined with the *phi* coefficient of association between species and site groups [37]. The size of site groups was equalized and the statistical significance of the association (*P*<0.05) assessed with Fisher’s exact test performed in the JUICE program [38]. Dominant species were defined as those having a percentage cover higher than 50% in at least 3% of plots.

The species richness of each forest type was expressed as the number of species (all and individual life-forms) in 100-m^2^ plots (a measure of local species richness or alpha diversity) and as sample-based rarefaction curves (a measure of total species richness or species pool) [39]. The curves were computed as means of 9999 sample-based species accumulation curves that resulted from random ordering of all plots belonging to each forest type. The calculation was performed with the JUICE program [38].

We compared plant diversity, environment, stand structure and functional traits between forest types with ANOVA and Tukey post-hoc tests. Because assumptions of parametric tests were not always met, we used randomization procedures for statistical testing. The observed test statistic was compared with the null distribution of the test statistic obtained via Monte-Carlo resampling with 9999 permutations. Analyses were run using the R software [33].

## Results

**Figure 2:**
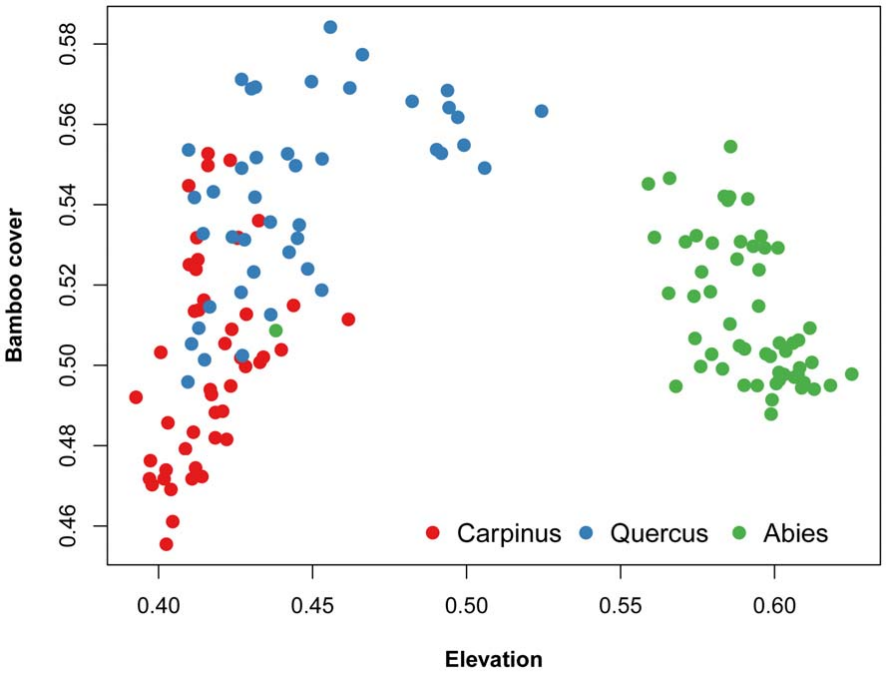
Multidimensional fuzzy set ordination diagram of forest vegetation plots from Halla Volcano, South Korea. The first axis represents a fuzzy elevation gradient from low elevation on the left to high elevation on the right. The second axis represents a fuzzy gradient from plots with low dwarf bamboo (*Sasa quelpaertensis*) cover on the bottom to plots with high bamboo cover on the top. Colors represent the three main forest vegetation types named after the dominant tree species.

The MFSO ordination revealed strong differences in forest composition between the cool areas at high elevation, where the coniferous *Abies koreana* and *Taxus cuspidata* trees dominated, and the warm areas at low elevation, where these cool temperate trees were replaced by warm temperate to subtropical trees of the *Carpinus* spp., *Quercus* spp., evergreen shrubs (*Ilex crenata*) and small trees (*Daphniphyllum macropodum*) (Figure 2). While the first axis represents the elevation gradient (r = 0.80, *P* = 0.001, 999 permutations), the second axis represents the gradient in dwarf bamboo cover (residual r = 0.03, *P* = 0.001, 999 permutations). The *Sasa quelpaertensis* bamboo cover clearly differentiates stands dominated by deciduous oaks (*Quercus mongolica*) from stands dominated by *Carpinus* and evergreen species (Figure 2). The correlation between plot distances in MFSO space and their original Bray-Curtis dissimilarities is 0.83, suggesting a highly effective ordination. Moreover, the plot configuration in constrained MFSO and unconstrained NMDS ordination is almost identical (Procrustes statistics m_12_ = 0.94, *P* = 0.001, 999 permutations). Elevation and cover of dwarf bamboo are therefore the main drivers of the vegetation patterns in the forests of Halla Volcano. The MRPP shows a highly significant (A = 0.157, *P* = 0.001, based on 999 permutations) differences in the species composition between the three forest types studied. The list of species diagnostic (indicator) and dominant for each forest type is provided in Table 2. The highest number of diagnostic species (46 taxa) was found in the *Abies* forests among herbs (17) and shrubs (9). The *Carpinus* forests had the highest number of diagnostic species among trees (9) and lianas (5), while the *Quercus* forests had the lowest number due to dominance of oak and maple trees, and understory dwarf bamboo.

**Table 2:** Vascular plant species composition of three forest types (Ca, *Carpinus*; Qu, *Quercus*; Ab, *Abies*) studied along an altitudinal gradient from 550 to 1940 m on Halla Volcano, Jeju Island, South Korea.

Forest type	Ca	Qu	Ab		Forest type	Ca	Qu	Ab
No. of plots	47	43	56		No. of plots	47	43	56
**Trees**					*Dryopteris expansa*	.	.	**34**
*Styrax japonicus*	**90**	33	.		*Phegopteris connectilis*	2	.	**30**
*Lindera erythrocarpa*	**74**	42	2		*Lycopodium obscurum*	.	.	**30**
*Daphniphyllum macropodum*	***70***	23	.		*Luzula plumosa*	.	.	**28**
*Ilex macropoda*	**47**	33	4		*Athyrium vidalii*	2	.	**29**
*Quercus serrata*	**43**	2	.		*Lycopodium clavatum*	.	.	**21**
*Cornus controversa*	**38**	19	4		**Forbs**			
*Lindera obtusiloba*	**34**	2	.		*Viola albida v. chaerophylloides*	**38**	21	2
*Neoshirakia japonica*	**32**	2	2		*Hosta clausa v. normalis*	**19**	.	4
*Meliosma myriantha*	**19**	.	.		*Asarum maculatum*	64	**93**	32
*Carpinus laxiflora*	***89***	***67***	2		*Maianthemum bifolium*	2	2	**77**
*Quercus mongolica agg.*	*38*	***81***	25		*Asarum sieboldii*	.	20	**71**
*Acer pseudosieboldianum*	83	**95**	21		*Ligularia fischeri*	31	16	**61**
*Carpinus tschonoskii*	*53*	***60***	.		*Parasenecio auriculatus agg.*	19	21	**54**
*Cornus kousa*	49	**58**	4		*Viola grypoceras*	.	.	**45**
*Abies koreana*	.	***19***	***98***		*Solidago virgaurea agg.*	.	.	**52**
*Prunus maximowiczii*	21	44	**73**		*Clintonia udensis*	.	.	**43**
*Sorbus commixta*	2	19	**57**		*Thalictrum tuberiferum*	2	2	**41**
*Betula ermanii*	.	.	**38**		*Galium kamtschaticum*	.	.	**39**
*Magnolia sieboldii*	4	5	**21**		*Fallopia forbesii*	.	.	**39**
**Shrubs and subshrubs**					*Angelica polymorpha*	.	.	**34**
*Ilex crenata*	***91***	51	20		*Oxalis acetosella*	.	.	**23**
*Ardisia japonica*	**26**	7	.		*Circaea alpina*	.	.	**20**
*Rhododendron mucronulatum*	2	.	**50**		*Potentilla stolonifera*	.	.	**20**
*Berberis amurensis v. quelpaertensis*	.	.	**43**		*Pternopetalum tanakae*	.	.	**18**
*Lonicera sachalinensis*	.	.	**41**		*Primula jesoana*	.	.	**18**
*Symplocos coreana*	.	9	**36**		*Geranium shikokianum*	.	.	**14**
*Vaccinium japonicum*	.	.	**36**		**Non-diagnostic species recorded in >25% of plots**			
*Chimaphila japonica*	.	2	**34**		*Smilax china*	70	67	18
*Weigela florida*	.	.	**30**		*Taxus cuspidata*	70	84	91
*Lonicera tatarinovii v. leptantha*	.	.	**18**		*Disporum smilacinum*	66	53	20
*Rhododendron yedoense v. poukhanense*	.	.	**16**		*Schizophragma hydrangeoides*	53	51	29
**Lianas**					*Hydrangea petiolaris*	13	23	41
*Hedera rhombea*	**38**	5	.		*Arisaema amurense*	47	30	23
*Codonopsis lanceolata*	**38**	12	.		*Carex ciliato-marginata*	43	30	18
*Akebia quinata*	**34**	2	.		*Dryopteris crassirhizoma*	45	33	52
*Trachelospermum asiaticum*	**30**	7	.		*Desmodium podocarpum agg.*	40	30	.
*Parthenocissus tricuspidata*	**19**	2			*Viburnum wrightii*	36	33	4
*Clematis koreana*	.	.	**38**		*Vaccinium hirtum v. koreanum*	30	26	9
**Grasses and sedges**					*Smilacina japonica*	30	14	4
*Oplismenus undulatifolius*	**34**	7	.		*Ainsliaea apiculata*	30	14	2
*Sasa quelpaertensis*	*70*	***100***	*62*		*Smilax sieboldii*	30	16	34
*Carex humilis agg.*	.	2	**55**		*Viola hondoensis*	30	21	2
*Carex erythrobasis*	5	5	**50**		*Viburnum erosum*	30	21	2
*Calamagrostis arundinacea*	.	.	***30***		*Arisaema ringens*	28	35	5
*Luzula plumosa*	.	2	**29**		*Prunus sargentii*	23	19	.
*Carex lanceolata*	.	.	**14**		*Viburnum furcatum*	26	30	29
**Ferns**					*Ophiopogon japonicus*	23	26	.
*Polystichum tripteron*	**21**	5	.		*Pourthiaea villosa*	15	28	18
*Huperzia chinensis*	26	16	**75**		*Hepatica asiatica*	11	16	29
*Huperzia serrata*	5	19	**63**		*Mitchella undulata*	6	19	27
*Athyrium yokoscense*	.	5	**45**		*Lepisorus ussuriensis*	2	23	27

Numbers are percentage frequencies of species occurrence; dots indicate absence. For trees, occurrences in both canopy and understory are counted. Diagnostic species with the phi coefficient of association > 0.3 are in bold. Dominant species (percent cover 50% in at least 3% of relevés) are in italic.

**Figure 3:**
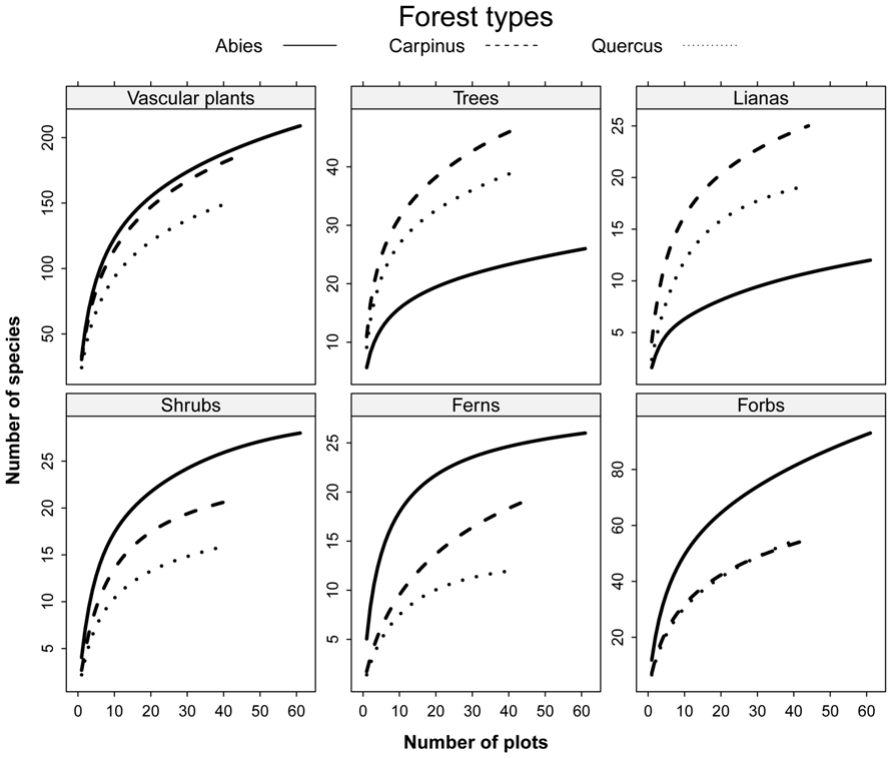
Sample-based rarefaction curves showing an increase in the number of species encountered in three forest types on Halla Volcano, South Korea with growing number of plots for all vacular plants and individual life forms.

When the forest types were compared in terms of local species richness, the lowest numbers of plant species were found in the *Quercus* forests, intermediate ones in the *Carpinus* forests and the highest in the *Abies* forests (Table 1). The opposite was found for the bamboo cover, being the highest in the *Quercus* forests, and less developed in both the *Carpinus* and *Abies* forests (Figure 2 and Table 1). With increasing bamboo cover, the species richness of vascular plants decreased (Table 1). A majority of species displayed an affiliation towards forests with low *Sasa* bamboo cover (Table 2). The species richness of forbs, grasses and shrubs were lowest in *Quercus* forests with high bamboo cover. The total species richness of larger areas estimated from the sample-based rarefaction curves (Figure 3) followed the same pattern as the local richness, with the fewest vascular plant species in the *Quercus* forests and the highest in the *Abies* forests. From 313 vascular plant taxa recorded within the 146 surveyed plots, 189 were found in the *Carpinus* forests, 149 in the *Quercus* forests, and 202 species in the *Abies* forests. The rarefaction curves estimated for individual life-forms revealed the highest number of shrubs, ferns and forbs species in the *Abies* forests and the lowest in the *Quercus* forests, while species richness of trees and lianas was highest in the *Carpinus* forests and lowest in the *Abies* forests (Figure 3). High spatial turnover in species composition (beta diversity), indicated by steep rarefaction curves, was found for forbs in the *Carpinus* and *Quercus* forests, whereas smaller beta diversity was found for tree species in the *Abies* forests, and fern species in the *Abies* and *Quercus* forests.

**Figure 4:**
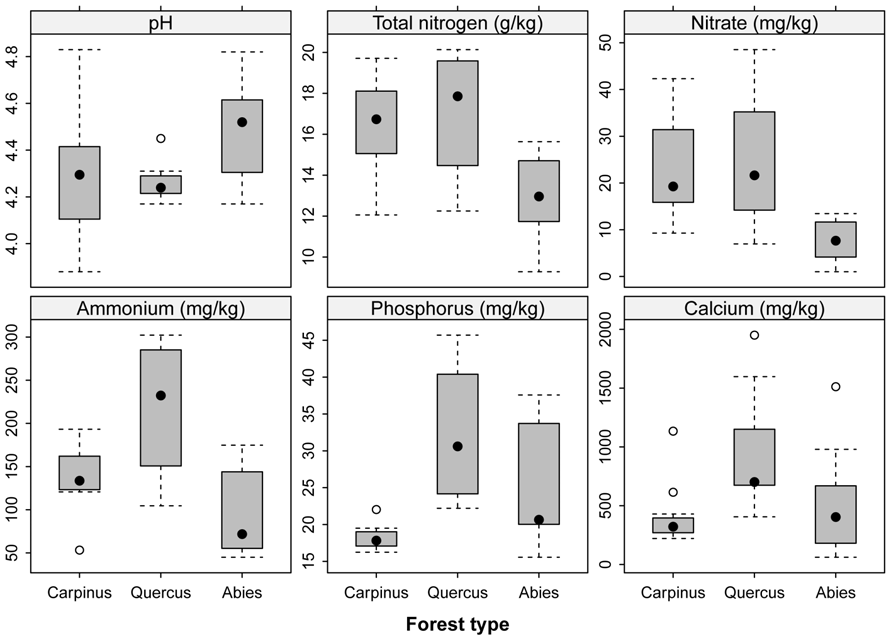
Comparison of soil reaction and concentration of soil nutrients between three forest types on Halla Volcano, South Korea. Boxes represent 25–75% of values, black dots medians, whiskers 1.5 interquartile ranges, and open dots outliers. For significant differences, see Table 1.

The forest types also differ in soil chemistry (Table 1). The soils in *Abies* forests are low in plant available nitrogen and base cations (Mg, Ca), while broad-leaved oak forests have a higher content of these elements (Figure 4). Concentration of plant available phosphorus was highest in the *Quercus* forests and lower in both the *Abies* and *Carpinus* forests. However, we did not find statistical differences in total soil nitrogen and soil reaction (Table 1).

**Figure 5:**
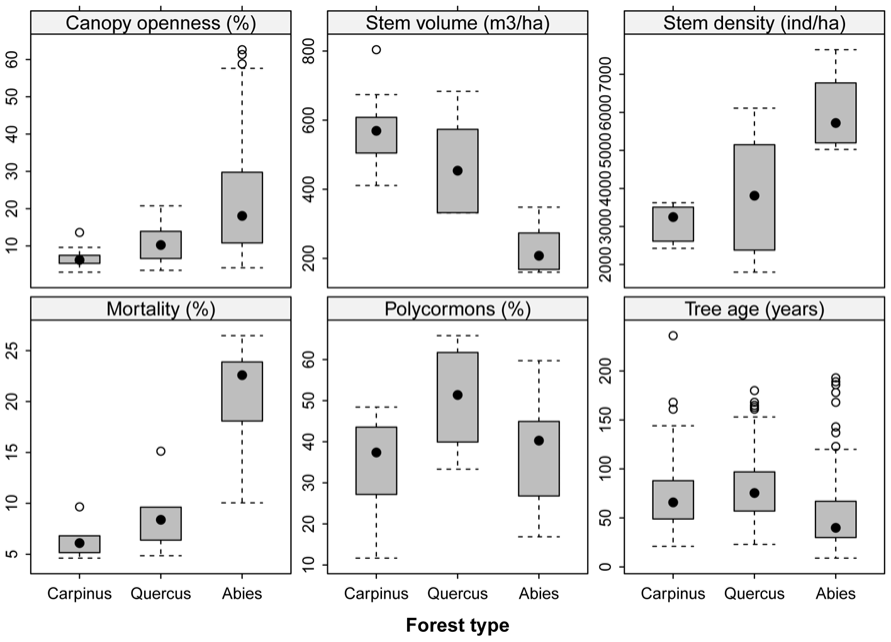
Comparison of stand characteristics between three forest types on Halla Volcano, South Korea. For results of multiple comparison tests, see Table 3.

**Figure 6:**
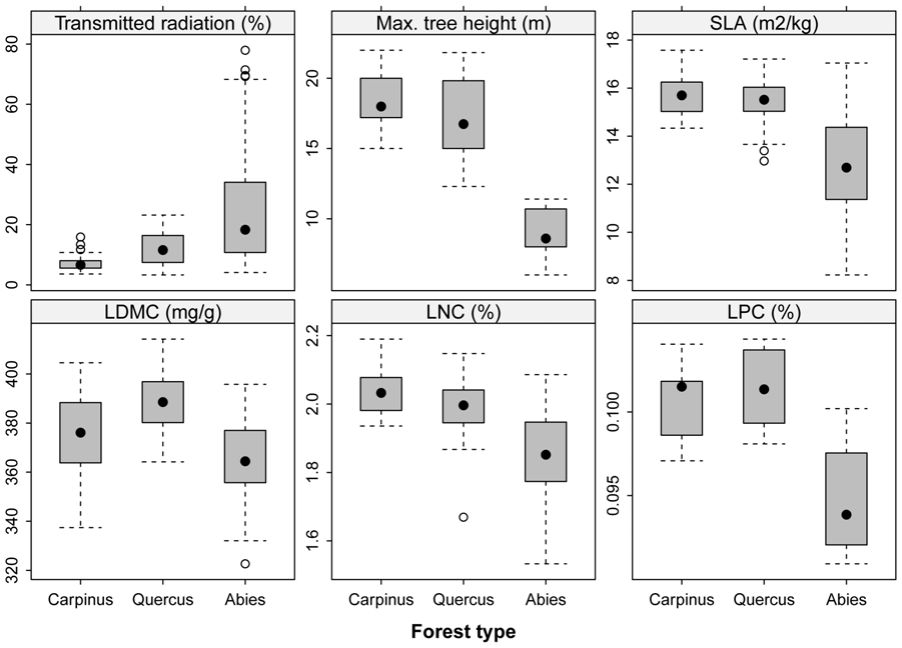
Comparison of transmitted direct radiation, maximum tree height, specific leaf area (SLA), leaf dry matter content (LDMC) and leaf N and P concentrations (LNC, LPC) of woody species among three forest types on Halla Volcano, South Korea. For significant differences, see Table 3.

There was also a remarkable difference in stand structure and functional traits of woody species between the two broadleaved deciduous forests and the *Abies* forests (Table 3 and Figure 5). The stem density was highest in the *Abies* forests, but trees had a smaller stem diameter and height and suffered higher mortality, resulting in the transmitted direct and diffuse radiation and canopy openness being highest in the *Abies* forests and the lowest in the *Carpinus* forests (Figure 5). Stem volume was high and not significantly different between the *Carpinus* and *Quercus* forests, but it was lower in the *Abies* forests. The proportion of multi-stemmed trees was highest in the *Quercus* forests, about fifty percent on average, and lower in both the *Abies* and *Carpinus* forests (Figure 5). The age of the studied trees ranged from 9 to 236 years, with a mean of 59 years. Trees in the *Abies* forests were younger, but had wider annual tree-ring increments than those in the *Carpinus* and *Quercus* forests (Table 3). Community-weighted means for LNC and SLA were highest in the *Carpinus* forests, intermediate in the *Quercus* and lowest in *Abies* forests, while LPC and LDMC peaked in the *Quercus* forests (Figure 6).

**Table 3:** Comparison of stand characteristics and functional traits of woody species between three forest types on Halla Volcano, South Korea.

Forest type	units	Carpinus	Quercus	Abies
Stem basal area	(m^2^ ha^–1^)	46.5^a^	48.6^a^	47.4^a^
Stem volume	(m^3^ ha^–1^)	595.0^a^	471.5^ab^	231.5^b^
Stem density	(ind. Ha^–1^)	3084^a^	3844^ab^	6075^b^
Proportion of dead stems	(%)	6.6^a^	9.6^a^	20.4^b^
Proportion of multi-stemmed trees	(%)	33.6^a^	50.6^b^	37.7^ab^
Mean tree age	(year)	73^a^	82^a^	51^b^
Maximum tree age	(year)	168^a^	236^a^	193^a^
Tree-ring width	(mm)	1.04^a^	1.04^a^	1.24^b^
Mean tree height	(m)	5.7^a^	5.4^a^	3.2^b^
Max tree height	(m)	18.4^a^	15.7^a^	8.4^b^
Mean DBH	(cm)	9.49^a^	9.5^a^	8.4^b^
Max DBH	(cm)	75.2^a^	94.9^a^	44.9^b^
Transmitted direct radiation	(%)	7.5^a^	12.3^b^	25.0^c^
Transmitted diffuse radiation	(%)	7.12^a^	11.68^b^	24.18^c^
Canopy openness	(%)	6.4^a^	10.5^b^	22.3^c^
Specific leaf area	SLA (m^2^ kg^–1^)	15.7^a^	15.4^a^	12.7^b^
Leaf dry matter content	LDMC (mg g^–1^)	376^a^	388^b^	364^c^
Leaf nitrogen concentration	LNC (%)	2.03^a^	1.98^b^	1.84^c^
Leaf phosphorus concentration	LPC (%)	0.100^a^	0.101^a^	0.094^b^

Means sharing the same lowercase superscript letter are not significantly different at *P* < 0.05 (ANOVA followed by Tukey post- hoc tests).

## Discussion

This paper attempts to improve our knowledge about paleoenvironmental changes in the southern part of the Korean Peninsula by comparing modern analogues of major forest types representing the Late Pleistocene-Holocene transition. These three forest types were studied on the Jeju Island, and we interpret them as a temporal sequence of postglacial colonization in concert with recent paleoecological data [18], [19], [21]. We used a comparative space-for-time substitution approach, which is often the only method available in a modern landscape. A basic assumption in this approach is that the different forest types are preserved close to each other under similar environmental conditions so as to avoid a confounding effect of different bedrock types and soil reactions resulting in different species pools. Such precondition is hardly met in mainland Korea because the late-Pleistocene coniferous forests along with alpine tundra vegetation largely retreated owing to the early Holocene spread of oak species [21], and because the mid-Holocene oak-hornbeam lowland forests were largely replaced by conurbation and paddy fields. We acknowledge that ecological inference from modern analogues using a space-for-time approach is not without risk as paleonvironments that are not analogous to any present situation were common in the past [6], [40]. At the same time, we believe that carefully interpreted analogues can significantly contribute to the ecological understanding of past environments [7], particularly when based on paleoecological evidence from macrofossil and pollen records [18], [19], [21].

The slopes of Halla Volcano (Hallasan) provide perhaps the best modern analogue of the Korean late-glacial to mid-Holocene forests, because here the deciduous broad-leaved forests of north Korean cool-temperate distribution meet with evergreen to deciduous forests of south Korean warm-temperate to subtropical distribution, and finally with postglacial refugia of coniferous forests with strong affinity to the cold maritime mountains of northeastern Asia [41]. The long-term persistence of different forest types on Jeju Island seems to be a result of different, often conflicting forces. Proximity of ocean and in particular of warm Tsushima Current (branch of the Kuroshio current), bringing warm and moist weather [11], ensured continuous presence of warm-temperate forest refugia during the LGM. While the lowland areas on the island provided a glacial refugium for broad-leaved trees during the coldest periods, the high mountain areas were a postglacial refugium for subalpine coniferous forests during the warmest periods of the Holocene. A gradual isolation associated with postglacial transgression and sea-level rise [18], and frequent summer typhoons and severe winters on Halla Volcano, preserved glacial floristic elements, including *Abies* subalpine forests and periglacial features such as thufur grassland vegetation on the top of Halla Volcano [24]. The present occurrence of several arctic-alpine species on Halla Volcano at the world's southernmost limit of their distribution (e.g. *Diapensia lapponica* subsp. *obovata*) [23], and of other species at the northernmost limit of their range in East Asia (e.g. *Daphniphyllum macropodum*), further supports the idea of plant refugia on this island.

Our results indicate that transition from the late-glacial coniferous forests to the early-Holocene broad-leaved deciduous forests was associated with important changes in ecosystem nutrient status: available soil nitrogen and phosphorus increases, as well as calcium and magnesium, which indicate soil enrichment with cations. The higher nutrient concentrations found in the uppermost soil layer of 1–5 cm in the mixed oak forests suggest a faster decomposition of the deciduous litter than of conifer needles, and hence increased nutrient cycling in deciduous forests [42]. This is corroborated by the higher nutrient content in leaf tissues of dominant woody species in the *Carpinus* and *Quercus* forests (Figure 6). It was probably the spread of woody species such as maple, hornbeam, and cherry that enhanced nutrient cycling in the early Holocene forests because of faster decomposition of leaf litter and its translocation below-ground. Similar changes in soil chemistry of postglacial forests were described from paleoecological data in Hungary [43] as well as from modern analogues of European Holocene forests in the southern Ural [7]. The trend of increasing soil N, P and cation contents can be reversed by a spread of evergreen broad-leaved trees and shrubs because their tough leaves are decomposed slower [44], [45]. This is indicated by decreased P, Ca, Mg concentrations in the uppermost soil layer of the *Carpinus* forests, where evergreen shrubs, small trees and lianas constitute the major component of the subcanopy layers (Table 1 and Figure 4).

The early Holocene changes in ecosystem nutrient status associated with the replacement of conifers by mixed oak forests had probably a significant effect on plant diversity. However, we suggest that the *Quercus* forests of the Halla Volcano usually have only about 5–10 vascular plant species per 100 m^2^ in their herb layer because of the higher degree of *Sasa* bamboo cover, whereas the *Abies* forests usually harbour 20–30 species in the same area.

Of the three forest types studied, the *Abies* forests had the highest herbaceous and total species richness. This can be explained by the lack of bamboo dominance in the understory and also by the life-history of *Abies koreana.* This light-demanding tree grows quickly by producing wide annual tree-ring increments (Table 3). This creates low wood density and makes its stem prone to wind-breakage and uprooting. High mortality in adult trees (Figure 5) creates canopy gaps that harbor light-demanding herbs and increase total diversity.

The most probable reason for the lower species richness in the *Quercus* forests is therefore shading by bamboo undergrowth and the thick layer of its litter precluding seed germination [46]. Dwarf bamboos are common understory dominants in mixed oak forests in NE Asia [41], where they form nearly continuous cover (often over 1 m in height), which effectively suppresses other plants. Hence, small numbers of herbaceous species and tree saplings are a characteristic feature of these forests, which are often confined to raised microsites such as fallen logs, stumps, and mounds created by tree falls [46], or, in case of woody species, reproduction hinges on vegetative propagation (stem-base sprouting and branch layering) leading to multi-stemmed polycormons (Figure 5). An alternative explanation for the lower species richness in the *Quercus* forests is that the spread of deciduous trees and shrubs in the early Holocene led to an increase in phosphorus and base cation availability. This could support more competitive tree species with denser canopies and prevent the establishment of light-demanding, competitively weak understory plants.

However, we suggest that the invasion of dwarf bamboo was more harmful for rich coniferous forests than the postglacial spread of oak species. The Late Pleistocene dynamics of dwarf bamboo were until recently impossible to reconstruct, as pollen data lack the resolution necessary to identify different grass taxa [47]. Recent studies based on opal phytolith analysis from southern Japan (200 km east of Jeju Island) indicate a continuous presence of dwarf bamboos over the last 30 ka, with a decline in response to the cool climate during the LGM (22-16 Ka), followed by a rapid increase after 13.5 ka [48], [49]. Thus, the dramatic decline of the light-demanding herbaceous species, and consequently also a decline of the total vascular plant diversity was probably due to an early Holocene dwarf bamboo invasion rather than spread of oak species. This idea is supported by evidence from temperate Europe where postglacial oak invasion into coniferous forests did not substantially reduce the rich pool of herbaceous species evolved and assembled over the long time period of the Pleistocene [7]. It was not until the spread of strongly shading tree species, hornbeam and beech, that a rich herb layer with light-demanding species was replaced by a smaller number of shade-tolerant forest herbs. Oaks are mostly light-demanding species, requiring large stand disturbances to successfully regenerate [46], but then they gradually overgrow other species to form the upper, more or less even-aged and relatively sparse canopy [50]. This provides enough light for the development of a rich herb understory. When lacking dense bamboo the Korean *Quercus mongolica* forests can harbour 30–35 species per 100 m^2^ in their herb layer [51]. This is three times more than in the same forest type where bamboo dominates.

In the *Carpinus* forests, the loss of light-demanding species caused by canopy shading and dense bamboo understory was partly compensated by an increase in shade-tolerant and often evergreen trees, shrubs and lianas. However, the pool of these species is much smaller than that of light-demanding herbs, and hence the total species richness is lower, both locally and in the whole area of the *Carpinus* and *Quercus* forests. The strongly shading tree species dominating the hornbeam forests have higher leaf tissue N and P concentrations and smaller leaf dry matter content (Figure 6). This enhanced litter decomposition and nutrient cycling and in turn favored the selection of highly competitive species in the shrub layer such as *Daphniphyllum macropodum, Ilex crenata, Meliosma myriantha, Neoshirakia japonica* and *Hedera rhombea* (Table 2). This further reduced available light and caused almost complete disappearance of understory herbs, including dwarf bamboo.

### Conclusions

Past diversity changes can be better understood using ecological knowledge from mountain refugia, where relict communities still exist close to communities which succeeded them elsewhere. Halla Volcano in Jeju Island harbors three forest types which represent a temporal sequence in which plant communities replaced each other during the Late Pleistocene-Holocene transition. We propose that the late-glacial open-canopy conifer forests in the southern part of the Korean peninsula were rich in vascular plants, in particular of light-demanding herbs. We have demonstrated that very probably, the postglacial oak invasion into coniferous forests did not substantially reduce this diverse pool of herbaceous species, because oak canopy provides relatively enough light to sustain a rich herb understory. The decrease in species richness was probably caused by an early Holocene spread of single species, strongly competitive *Sasa* understory bamboo. The subsequent spread of maple and hornbeam caused canopy closure and an almost complete disappearance of understory herbs including *Sasa* bamboo. This postglacial species replacement was associated with important changes in plant functional traits affecting ecosystem processes such as litter decomposition, nutrient cycling and wood production.
